# Actin and Actin-Associated Proteins in Extracellular Vesicles Shed by Osteoclasts

**DOI:** 10.3390/ijms21010158

**Published:** 2019-12-25

**Authors:** L. Shannon Holliday, Lorraine Perciliano de Faria, Wellington J. Rody

**Affiliations:** 1 Department of Orthodontics, College of Dentistry, University of Florida, Gainesville, FL 32610, USA; 2 Department of Biomaterials and Oral Biology, School of Dentistry, University of São Paulo, São Paulo 01000, Brazil; LPercilianodeFaria@dental.ufl.edu; 3 Department of Orthodontics and Pediatric Dentistry, Stony Brook University School of Dental Medicine, Stony Brook, NY 11794, USA; Wellington.Rody@stonybrookmedicine.edu

**Keywords:** exosome, microvesicle, microfilament, integrins, bone remodeling, myosins, actin-related protein, proteomics, extracellular vesicles

## Abstract

Extracellular vesicles (EVs) are shed by all eukaryotic cells and have emerged as important intercellular regulators. EVs released by osteoclasts were recently identified as important coupling factors in bone remodeling. They are shed as osteoclasts resorb bone and stimulate osteoblasts to form bone to replace the bone resorbed. We reported the proteomic content of osteoclast EVs with data from two-dimensional, high resolution liquid chromatography/mass spectrometry. In this article, we examine in detail the actin and actin-associated proteins found in osteoclast EVs. Like EVs from other cell types, actin and various actin-associated proteins were abundant. These include components of the polymerization machinery, myosin mechanoenzymes, proteins that stabilize or depolymerize microfilaments, and actin-associated proteins that are involved in regulating integrins. The selective incorporation of actin-associated proteins into osteoclast EVs suggests that they have roles in the formation of EVs and/or the regulatory signaling functions of the EVs. Regulating integrins so that they bind extracellular matrix tightly, in order to attach EVs to the extracellular matrix at specific locations in organs and tissues, is one potential active role for actin-associated proteins in EVs.

## 1. Introduction

**Figure 1:**
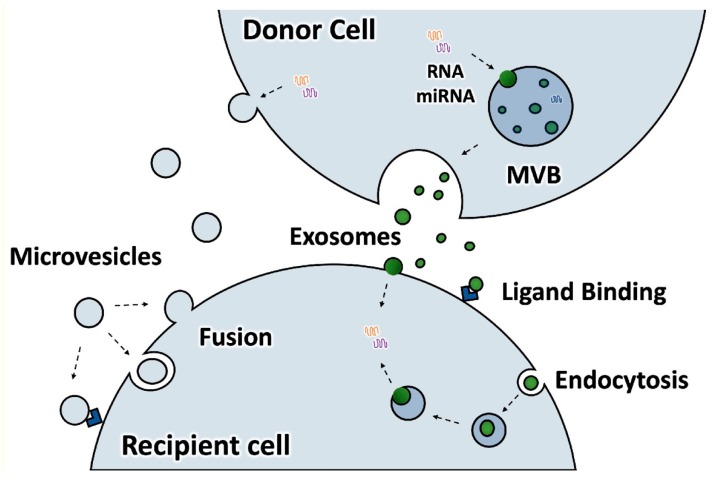
Extracellular vesicles include exosomes which are derived from multivesicular bodies (MVB) and microvesicles (ectosomes) which bud directly from the plasma membrane. Both may bind surface receptors of target cells to stimulate signaling pathways, or to fuse with the plasma membrane or membranes of endocytic compartments. Fusion releases their luminal contents into the cytosol of the target cell, and membrane proteins into either the plasma membrane or endocytic membrane.

Extracellular vesicles (EVs) are 30–150 nm in diameter vesicles that are released by eukaryotic cells and function in intercellular signaling [1,2]. The term EVs encompasses exosomes and microvesicles (Figure 1) [3]. Exosomes develop as inward buds into endocytic compartments, which pinch off into the lumen of the compartment, resulting in the formation of multivesicular bodies. Multivesicular bodies can then fuse with the plasma membrane to shed the exosomes from the cell. Microvesicles bud off directly from the plasma membrane. The two types of EVs have similar size, composition, and regulatory functions and are difficult to distinguish in extracellular vesicle populations, although some articles suggest that microvesicles may be on average larger and may have some differing components [4]. In addition, some non-vesicular particles are probably often isolated in EV preps, including exomeres and lipoproteins [5]. Unless the type of vesicle being studied is known, which is usually not the case at the present time, the term EVs is preferred [3]. 

Exosomes were first identified and characterized due to their role in the removal of the transferrin receptor from reticulocytes as they differentiated [6,7]. For many years, exosomes were mostly thought of as “garbage bags”, although evidence that EVs could present antigen appeared during the 1990s [8]. In 2007, landmark articles showed that exosomes carried mRNAs and microRNAs, and could fuse with target cells to introduce the functional RNAs into the cytosol [9,10]. The concept of EVs being able to regulate target cells acting at different regulatory levels stimulated the EV field. Subsequently, much evidence has accumulated that by transferring microRNAs, EVs modulate target cell protein expression. For example, two groups reported that microRNA 214-3p is found in EVs from osteoclasts, and is transferred to osteoblasts, where it inhibits osteoblast formation by reducing the expression of regulatory proteins [11,12]. Despite the plethora of articles supporting the hypothesis that microRNAs in EVs are crucial to their regulatory function, some studies have cast doubt on whether sufficient numbers of microRNAs are present in EVs to suppress mRNA translation [13].

For EVs to bind and stimulate a target cell, either from the outside through traditional signal transduction pathways, or after fusing, the EVs must interact with the cell. Osteoclast EVs serve as a model for the sorts of interactions and regulation that have been found in EVs in general.

In osteoclasts, three potential modes of interaction have been identified, namely semaphorin 4D in EVs binding plexin-B1 on osteoblasts [11], ephrin-B2 in EVs binding ephB4 [12], and receptor activator of nuclear factor kappa B (RANK) in EVs binding RANK-ligand (RANKL) [14]. Semaphorin 4D and ephrinB2 were reported to be osteoclast-derived signaling factors, before their detection in EVs [15,16]. Both were found to tether EVs to the surface of osteoblasts prior to the fusion that delivers microRNA-214-3p from the EVs lumen to the cytosol of the osteoblast. 

RANK is part of the central signaling pathway in bone remodeling [17,18]. RANKL, which is found on osteoblasts and osteocytes, binds RANK on the surface of osteoclasts to stimulate a signaling pathway, resulting in the activation of nuclear factor kappa B and nuclear factor of activated T cells 1 signaling. These pathways are required for osteoclasts to differentiate from multipotent hematopoietic precursors to osteoclasts, and for the bone resorbing activity by mature osteoclasts. A third protein, osteoprotegerin, is a soluble protein secreted by osteoblasts that binds RANKL and serves as a competitive inhibitor of binding between RANKL and RANK. The detection of RANK-containing EVs (RANK-EVs) suggested immediately the possibility that they might function like osteoprotegerin as competitive inhibitors of binding between RANKL and cellular RANK. Consistent with this idea, when added to calcitriol-stimulated mouse marrow, in which calcitriol stimulates the coordinated differentiation of osteoblasts, which produce RANKL, and osteoclasts, RANK-EVs reduced osteoclast formation [14]. Quantitative assessment of the data, however, suggested that there were likely too few RANK-EVs to trigger the observed reduction in osteoclast numbers by simple competitive inhibition, and another regulatory mechanism was likely in play [19].

A solution was presented when it was shown that RANK-EVs stimulate a reverse RANKL signaling pathway through mammalian target of rapamycin and runt-related transcription factor 2, which drive cells of the osteoblastic lineage toward bone formation [20]. This idea is also consistent with findings that osteoblasts and osteoclasts are rarely in direct contact in vivo. In addition, increasingly strong evidence shows that osteocytes, terminally differentiated cells of the osteoblastic lineage which are buried in the bone, are the source of most of the RANKL that stimulates bone resorption in vivo [21,22,23,24]. Hence, RANK-EVs produced by osteoclasts in vivo would be free to bind RANKL on osteoblasts to stimulate bone formation, thus providing an elegant mechanism for coupling bone resorption to bone formation [20].

Although this provides a satisfying explanation for regulation by RANK-EVs, it leaves open the question of whether these EVs fuse with osteoblasts and whether other components, like microRNAs, may be present in RANK-EVs. Another crucial question regarding RANK-EVs is how their shedding is regulated. More RANK-EVs were shed from osteoclasts resorbing bone compared with dentine or when they were quiescent on plastic [25]. Understanding the function of regulatory EVs from osteoclasts requires more complete knowledge of their composition.

## 2. Proteomics Show that EVs Shed by Osteoclasts Are Rich in Actin and Actin-Associated Proteins

**Figure 2:**
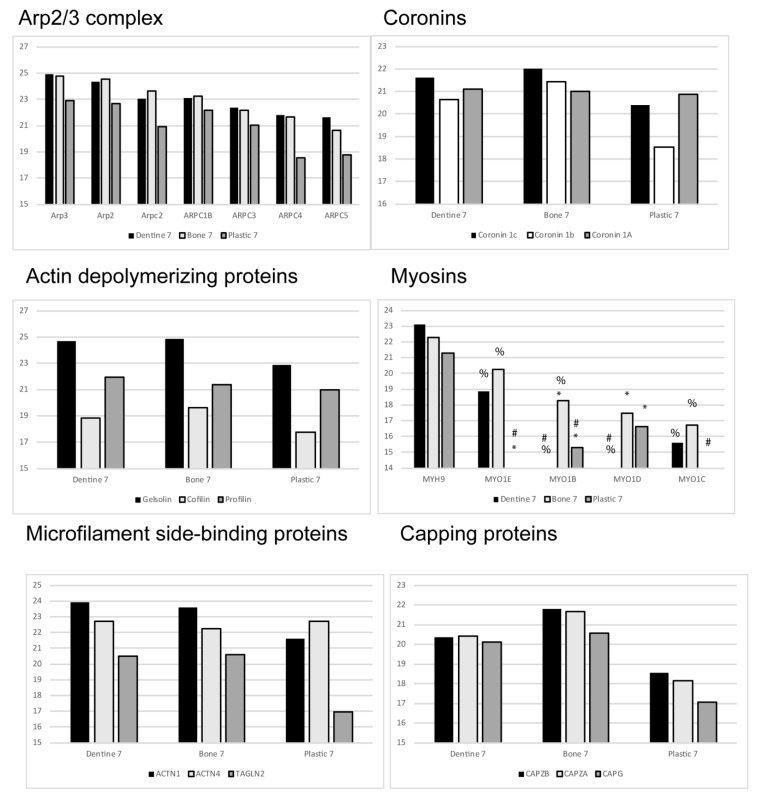
The relative abundance of the actin associate proteins discussed in the current manuscript. These numbers are normalized to actin in extracellular vesicles from conditioned media form osteoclasts resorbing bone at Days 4–7. The raw Day 4–7 data were published previously [25]. Abbreviations: MYH (myosin heavy chain), MYO (myosin), ACTN (alpha-actinin), TAGLN (transgelin), CAP (capping protein). Statistical analysis was performed from Z-scores as described in reference 25. Proteins with Z-scores greater than 1.65 or smaller than 1.65 were considered significantly different. The following symbols are used; # indicates different from bone; % indicates different form plastic; * indicates different from dentine.

We performed two-dimensional, high resolution liquid chromatography/mass spectrometry of EVs isolated from osteoclasts resorbing bone, dentine or quiescent on plastic [25]. The morphology of osteoclasts on bone and dentine was indistinguishable, while those on plastic lacked actin rings and ruffled membranes. The quantitative and qualitative data we obtained identified actin as the most abundant protein of EVs resorbing bone, and various actin-associated proteins as being very abundant. The levels of actin associated proteins referred to in the manuscript, except integrins, which will be presented separately, are shown in Figure 2. These numbers are normalized to actin in EVs from bone resorbing osteoclasts.

**Figure 3:**
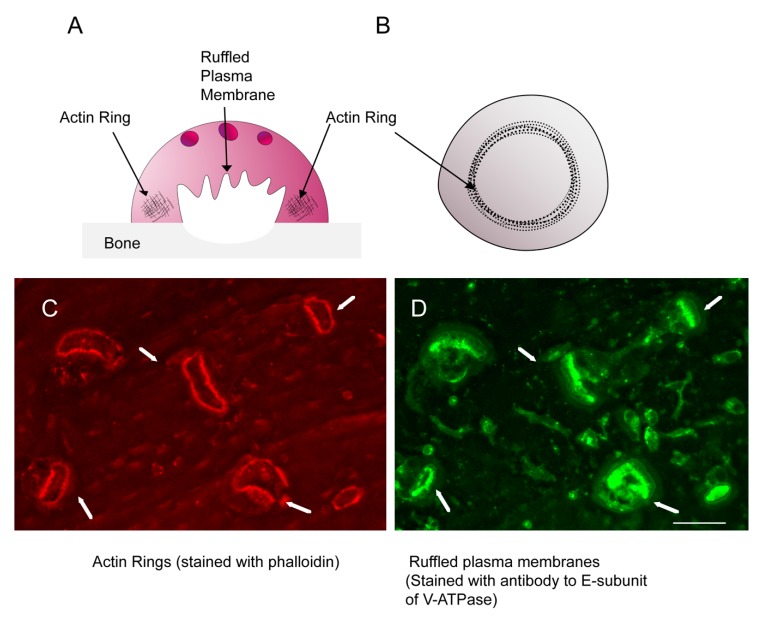
Osteoclasts are highly specialized cells that migrate through mineralized tissue. (**A**) A schematic of a resorbing osteoclast from the side. (**B**) Schematic of cell from side. (**C**) Immunofluorescence micrograph of actin rings stained with Texas Red-phalloidin (red). (**D**) Immunofluorescence micrograph of same cells stained with a rabbit polyclonal anti-E-subunit of V-ATPase antibody followed by an Alexa 488-tagged anti-rabbit IgG secondary antibody to detect ruffled plasma membranes (green). Arrows show examples of actin rings and ruffled membranes, with arrows pointing to same cells in the two panels. The scale bar for the micrographs is 25 μm.

Osteoclasts are highly specialized to resorb bone [26]. To do this, they form resorption compartments, each composed of an actin ring and a ruffled plasma membrane, or ruffled border (Figure 3). Actin rings are composed of arrays of podosomes (also called invadopodia) [27]. These dynamic structures are very similar to the podosomes found in other cell types [28], but to meet the needs of resorbing osteoclasts, they are joined by interconnecting actin filaments to form the cohesive actin ring [29]. The actin ring is associated with a very tight contact between the osteoclast membrane and bone, the sealing zone, which segregates an extracellular resorption compartment. The ruffle plasma membrane is surrounded by the actin ring and is packed with vacuolar H^+^-ATPase (V-ATPase), which pumps protons out of the cell into the resorption compartment [30,31]. This lowers the pH to about 5.0, which solubilizes bone mineral, and provides an environment where the acid cysteine proteinase, cathepsin K, which is secreted by the osteoclasts, degrades the organic matrix of the bone [32,33]. Because of the prominent role of the actin cytoskeleton in osteoclasts, it is perhaps not surprising that EVs would be loaded with abundant actin and associated proteins. Nevertheless, to understand extracellular vesicles, the potential roles of the most abundant components, which include actin and associated proteins, must be addressed. Even if they have no functional role, that too must be understood, and that would be a clue regarding the formation of EVs and their functions. As a first step in this process, in this article we will discuss what is found, and what is not found, in EVs and the questions that are raised.

In the discussion that follows, we will assume that actin and associated proteins are evenly distributed among EVs. This is unlikely to be the case. However, this simplification makes it easier to organize the quantitative data and given that actin and associated proteins are very abundant in EVs from various sources, it may be a reasonable approximation. It is possible that these proteins are concentrated in a subset of EVs, for example, into microvesicles and not exosomes. As we go through the different types of actin-associated proteins found in osteoclast EVs, we will provide a brief review of their known functions in cells and discuss how the specific proteins may be involved in the formation and/or function of EVs.

## 3. Possible Roles for the Actin Cytoskeleton in the Formation of EVs

As EVs form from the budding of a membrane either into an endocytic compartment or from the plasma membrane, the actin cytoskeleton may play a role in the process, and indeed may be included in EVs due to its role in EV formation. There is some data supporting this idea for the formation of microvesicles (also called ectosomes) [34]. The actin cytoskeletal protein filamin A was shown to be involved in regulating the incorporation of tissue factor into EVs [35,36]. It remains uncertain, however, whether elements of the actin cytoskeleton are required for EV formation. The endosomal sorting complexes required for transport (ESCRT) complex [37], Rab27 [38], and neutral sphingomyelinases [39,40] have all been implicated in EV formation.

**Figure 4:**
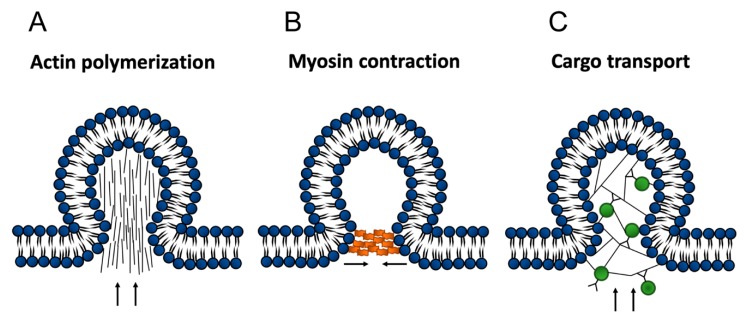
Three ways that the actin cytoskeleton might be involved in extracellular vesicle (EV) formation. (**A**) Microfilaments may push against membranes using force generated by polymerization of actin. (**B**) Myosin motors may materials to the site of EV formation for packaging. (**C**) Myosin contraction may play a role in the scission of the EV. In each of these cases, elements of the actin cytoskeleton may be trapped in the EVs as the result of the cytoskeleton’s involvement in the EV formation process. Arrows denote the direction filaments push into membrane in actin piloymerization, or the direction of cargo transport.

At least three ways can be envisioned by which the actin cytoskeleton could be involved in EV formation. Actin filaments may be involved by pushing against the membrane, harnessing the force generated by actin polymerization to form a bud [41,42,43]. Myosin mechanoenzymes could move components to sites where EVs are forming [44]. Actin–myosin contraction could be involved in sealing buds that form [45] (Figure 4).

Crawling motility is powered by force generated by actin polymerization to push against membranes [46], so the notion of actin polymerization pushing against membranes to facilitate bud formation is plausible. Likewise, myosins for delivering materials [47] and for the scission of membranes, as in cytokinesis where myosin II works in conjunction with elements of ESCRT [48], is well established. In addition, myosin contraction, downstream of ADP-ribosylation factor 6 (ARF6) signaling, has been implicated in the formation of microvesicles [49]. A conventional myosin II and ARF6 are detected in osteoclast EVs [25].

Testing these ideas is more difficult, since actin polymerization and myosins are involved in a wide array of cell processes. To formally test this idea likely will require use of a cell-free model of multivesicular body formation [50]. Ideally a model could be developed that incorporates a minimal number of purified components, in a similar way to that in which the force generation mechanism of actin polymerization has been characterized [51,52].

## 4. Are Microfilament Dynamics Possible in EVs?

Actin exists as either a 42 kD monomer or as polymers of the monomer called microfilaments [53]. Typically in cells, about half of the total actin is polymerized into microfilaments. Polymerization and depolymerization in the cytosol occur constantly and are tightly controlled [54]. Is actin polymerized or unpolymerized in EVs and do transitions between monomers and polymers occur? Filaments within subpopulations of EVs leading to non-spherical EVs, and perhaps dynamics of the filaments have been reported [55,56]. This provides some empirical support for the idea that dynamic cytoskeletal systems may exist in at least some EVs. As we will discuss, some, but not all, of the components normally associated with actin dynamics are detected in EVs. However, cytoskeletal dynamics in the cytosol of cells is tied to a high rate of adenosine triphosphate (ATP) hydrolysis [57]. There is evidence that ATP can be generated by glycolytic enzymes in certain types of EVs, prostasomes, and seminal plasma exosomes [58,59]. However, it has not been shown that the ability to generate ATP through glycolysis occurs in the lumen of EVs in general.

Actin can bind either ATP, ADP-Pi, or ADP. ATP-actin polymerizes much better and is the species that usually (or always) enters filaments in cells. Soon afterward, the actin-bound ATP is broken down, first to ADP-Pi-actin, then ADP-actin. This occurs as a result of its presence in the microfilament [57]. When filaments depolymerize, ADP-actin typically is released and it is then recharged into ATP-actin from the cytosolic free ATP pool by interaction with profilin, which binds actin monomers and causes a change in conformation that accelerates nucleotide exchange [60]. This in practice means conversion of ADP-actin to ATP-actin, since ATP is much more abundant in the cytosol than ADP.

EVs isolated by standard methods, including EVs from osteoclasts, have abundant glycolytic enzymes [25]. However, recent evidence suggests that at least a portion of the glycolytic enzymes normally detected in EV preparations are components of non-vesicular structures called exomeres [61]. Even if EVs are rich in glycolytic enzymes, no membrane transporters for glucose or other sugars were detected in osteoclast EVs [25]. It is possible that such transporters are present at low levels, which may be enough to supply glucose or other raw materials for glycolysis. Nevertheless, it has not yet been demonstrated that osteoclast EVs, or most other EVs, have the energy producing resources to support traditional actin dynamics. This also does not rule out some sort of non-traditional polymerization scheme. For example, high concentrations of ADP-actin will polymerize [62]. However, unless a mechanism for ATP generation or the import of ATP into EVs is identified, serious concerns about the plausibility of actin dynamics in EVs are warranted.

Finally, isolated EVs are typically stored in media lacking ATP or free sugars. This would mean the EVs that are usually studied by electron microscopy or nanoparticle tracking lack an energy source. It may be worth studying EVs under conditions where ATP might be available or could be, in principle, generated. 

One mechanism by which actin polymerization is triggered in cells is through the action of a class of proteins called formins [63]. None of this family were detected in EVs from osteoclasts.

The actin related protein 2/3 (Arp2/3) complex is the basis of the other general mechanism for triggering actin polymerization in cells. It contains one copy each of seven different proteins including Arp2 and Arp3, which are close relatives of actin [64,65,66]. Arp2/3 is activated to stimulate actin polymerization by interactions with members of the Wiskott–Aldrich syndrome protein (WASP) family of proteins [67]. WASP proteins are activated by Rho-class GTPases [54]. In osteoclast EVs, all of the elements of the Arp2/3 complex are very abundant and, in the stoichiometry, expected for intact complexes (Figure 2). We estimate about 1 copy per 7 actin monomers. Surprisingly, we only detected traces of two members of the WASP family of proteins, WASP and WAVE2. It is not clear that the abundant Arp2/3 complex could be activated to stimulate actin polymerization in EVs.

In principle, Arp2/3 complex could enter EVs independent of actin, associated with the “slow-growing” end of actin filaments, or as part of a branched actin network. The incorporation of the Arp2/3 complex into branched actin networks can involve interactions with cortactin and n-WASP (one of the WASP family) [68]. Cortactin is upregulated as osteoclasts differentiate, is expressed at high levels in mature osteoclasts, and it is required for the formation of actin rings [69,70,71]. However, it was not detected in osteoclast EVs, even in trace amounts. 

Three isoforms of coronin were abundant in osteoclast EVs [72]. Coronin binds and locks Arp2/3 into an inactive complex in the absence of preexisting filaments, but links Arp2/3 to existing filaments, and can either protect them from or enhance the activity of cofilin [73], a protein that binds to the side of actin filaments, disassembles filaments and binds actin monomers. It is abundant in osteoclast EVs (Figure 2). Coronin binds ATP-actin with 47-times higher affinity that ADP-actin. It protects microfilaments from cofilin when it is bound to ATP-actin but does not protect ADP-actin. As discussed above, it seems likely that most of the actin in EVs is ADP-actin. Therefore, although EVs may have initially have had polymerized actin in them, for example, if actin polymerization has a role in EV formation, very soon microfilaments would be expected to be disassemble, aided by the activities of cofilin, gelsolin, and profilin [74,75], which are abundant, unless they are protected by stabilizing proteins. Coronin may associate with Arp2/3 to maintain it in the inactive state. In this scenario, ADP-actin would be unlikely to spontaneously polymerize as it requires a higher concentration to polymerize than ATP-actin, and the abundant profilin and cofilin, which sequester actin from polymerization, would bind ADP-actin monomers and keep the free actin concentration low. It is worth considering that if such an EV was to fuse with a target cell, many of the necessary raw materials for actin polymerization would be locally enriched, requiring only the abundant free ATP in the cytosol, and a WASP family member to trigger rapid local polymerization. Such an event could mechanistically help explain the observation that EVs can contribute to persistent cell movement through extracellular matrix [76,77]. The fusion of EVs associated with the matrix with the migrating cell could provide concentrated packages of cytoskeletal components at the leading edge, in addition to signaling molecules.

Molecule interacting with CasL (Mical) catalyzes redox reactions using microfilaments as a substrate [78]. This makes the filaments more susceptible to depolymerization by cofilin [79]. Mical1 is present in osteoclast EVs.

Based on this analysis, it seems unlikely that actin assembly/disassembly cycles occur in EVs in the manner of the cytosol, at least in most EVs, most of the time. There is probably neither the ATP to support polymerization nor the regulatory proteins to promote polymerization. In addition, any new filaments assembled would be rapidly disassembled by depolymerizing proteins, unless they were protected and stabilized. As we will see, proteins that could protect and stabilize filaments are present. It is therefore possible that microfilaments enter EVs as the EVs are formed and are stabilized to remain polymerized in the EVs.

## 5. Stable Microfilaments in EVs?

Before examining whether stable microfilaments might exist in osteoclast EVs, it is worth considering the possible length of a microfilament that could be contained in an osteoclast EV, which are on average about 50 nm in outside diameter. Structurally, each monomer in a microfilament is associated with an increase in length of about 3 nm, and microfilaments do not bend very much [53]. This constrains the possible length of microfilaments in most osteoclast EVs to be no more than about 30 nm in length (10 monomers). Even in a large, 150 nm-diameter EV, the size limit is about 50 monomers. In cells, microfilaments often achieve lengths of a micron or more, and contain hundreds of monomers Unless microfilaments deform the EVs, only relatively short microfilaments are physically possible in EVs.

In cells, subsets of microfilaments are stable, depolymerizing slowly or not at all [41]. These microfilaments are bound by proteins that interact with ADP-actin in filaments and protect the filaments from the actions of depolymerizing proteins like cofilin. Proteins also stabilize microfilaments by reducing depolymerization through the binding of free filament ends and blocking actin dynamics at that end. Osteoclast EVs contain a number of abundant proteins from this category.

The following proteins that bind to, and block the fast-growing ends of filaments are abundant in osteoclast EVs, gelsolin, the alpha and beta subunits of capZ and capG [80]. The slow growing end of actin filaments has a higher critical concentration [53]. It is possible that capped stabilized filaments could be incorporated into EVs and stabilized as short filaments. Such short filaments would fit in the lumen of EVs and might be appropriate for stabilizing protein complexes that interact with and regulate the binding state of integrins, as will be discussed in more detail below. Vasodilator-stimulated phosphoprotein (VASP) is an end tracking protein that has also been reported to protect microfilaments from depolymerization by gelsolin [81]. It is detected in EVs with modest abundance.

Tropomyosins bind the sides of microfilaments and protect them from degradation by cofilin [82]. Different isoforms of tropomyosin provide differing levels of protection [83]. Three tropomyosins are detected in osteoclast EVs though none are abundant. Tropomyosin 3 is the most abundant of the three and provides the greatest protection against cofilin severing. Plastins, including l-plastin, which is abundant in the EVs, compete with tropomyosins and cofilin for binding the microfilaments. They synergize with cofilin, under some conditions, to displace tropomyosin [82].

Transgelin-2 is a microfilament-binding protein that protects microfilaments from cofilin-mediated depolymerization, and it is a tumor-suppressor [84,85,86,87,88]. This small protein is abundant in EVs, and in principle could be involved in stabilizing a significant amount of the actin as microfilaments. Little is known about transgelin-2 in osteoclasts or EVs.

## 6. Myosins in EVs

By far the most abundant myosin in osteoclast EVs is the conventional non-muscle myosin IIa (MYH9). This is a common myosin to be found in EVs from other sources and was identified as a potential exosomal biomarker for inflamed trigeminal satellite glial cells [89]. In osteoclasts, both myosin IIa and myosin IIb are expressed, and myosin IIa is associated with the actin rings. Myosin IIb is associated with the non-actin ring actin cytoskeleton and was detected in trace amounts in EVs from osteoclasts resorbing bone [90]. 

The unconventional myosin 1E was the second most abundant myosin detected. It has not been studied in osteoclasts but was recently identified as a regulator of phosphatidylinositol signaling and actin polymerization in posodomes in mouse embryonic fibroblasts [91]. Several other myosin I isoforms (myosin 1B, myosin 1D, myosin 1C, myosin 1F, and myosin 1G) were found in trace amounts. Likewise, traces of myosin Va, myosin IIb, myosin XIV, myosin XVIIIA, myosin XV, and myosin VIIB were detected. Myosins are large proteins. We detected 50–80 peptides from myosin IIa in our proteomic analysis. We only detected only 2–4 peptides from the trace myosins, usually in only one sample [25].

As with the discussion of actin dynamics, a major limiting factor of potential myosin activity in EVs is whether ATP for the motor would be present. If not, it would likely be locked in ATP-free rigor. The presence of abundant myosin IIa could implicate it in EV formation. It will be important to test whether myosin contraction is required for exosome or microvesicle formation.

## 7. Integrins and Regulators of Integrin Binding in EVs

Integrins in EVs have garnered considerable attention for their ability to connect EVs to specific sites in the extracellular matrix [92,93,94,95]. The activity of integrins in EVs has been linked to promoting metastasis of cancer cells and to pulmonary tissue destruction [76,96,97]. More generally, it is thought that integrins are at least partially responsible for observed organotropism [98,99]. For example, EVs collected from osteoclasts in cell culture, then injected into the tail vein of mice, were shown to preferentially hone to bone, and EVs from other types of cells to hone preferentially to their organ of origin [11]. An important mechanistic concern remains to be addressed. Integrins, which are composed of an alpha and beta subunit, in the plasma membrane can exist in three conformations, only one of which binds the extracellular matrix with high affinity [100]. These conformational states are regulated by proteins that bind directly or indirectly to the cytosolic (or in the case of EVs luminal) domain of the integrins. These include talin, filamin, vinculin, and l-plastin, which are all abundant in osteoclast EVs. 

**Figure 5:**
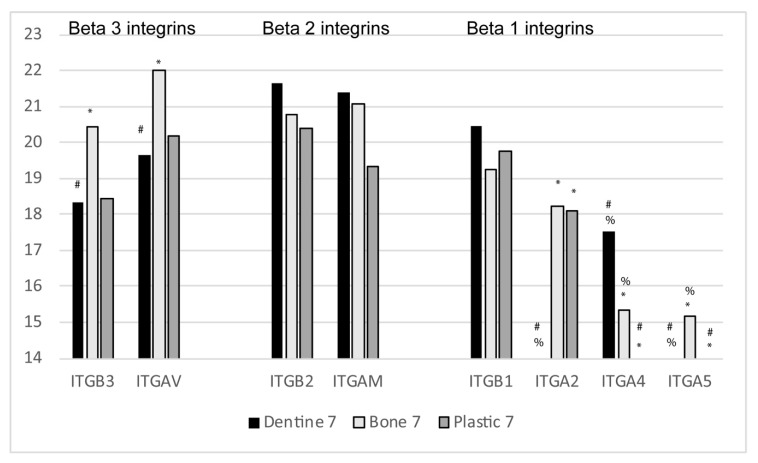
The relative abundance of the integrins (ITGB is integrin beta; ITGA is integrin alpha) found in osteoclast extracellular vesicles. AlphaV beta3 were found at higher levels in EVs from osteoclasts resorbing bone, compared with dentine or quiescent osteoclasts. Beta2 was found at higher levels in EVs from dentine. Our data suggest that beta1 integrin is found at higher levels in EVs from dentine. Alpha2 beta1 is probably found in all samples, although alpha2 was not detected in the EVs from dentine it was not detected in Day 7 dentine. Alpha4 beta1 may be found at higher levels in osteoclasts resorbing dentine and alpha5 beta1 may be more prominent in osteoclasts resorbing bone. Statistical analysis was performed from Z-scores as described in reference 25. Proteins with Z-scores greater than 1.65 or smaller than −1.65 were considered significantly different. The following symbols are used; # indicates different from bone; % indicates different form plastic; * indicates different from dentine.

The most abundant integrins found in osteoclast EVs are alphaV beta3, alphaM beta2, and alpha2 beta1 [25]. The proteomic experiment performed was designed to compare osteoclasts resorbing bone or dentine and osteoclasts quiescent on plastic. AlphaV beta3 was more abundant on EVs from osteoclasts resorbing bone than on those resorbing dentine, while the reverse was true for alphaM beta2 (Figure 5). Our data suggest that there is more beta2 than alphaM, it is likely that very small amounts of alphaL and/or alphaX, other partners of beta2, are also present, but too little to detect. AlphaM/X/L beta 2 have all been reported to be present in osteoclasts or in osteoclast precursors [101,102,103,104,105]. It is possible that EVs containing these integrins are from precursor cells in the culture and are being shed during osteoclast differentiation.

Three binding partners for beta1 integrin were found, alpha2, which was most abundant, alpha4 and alpha5. Interestingly alpha4 was more prominent in EVs from osteoclasts resorbing dentine while alpha5 was more prominent in EVs from osteoclasts resorbing bone. The binding specificity for the three partially overlaps but has key differences. Alpha2 beta1 binds collagen, laminin, and thrombospondin. Alpha4 beta1 binds thrombospondin, osteopontin, fibronectin, and mucosal vascular addressin cell adhesion molecule (MAdCAM). MAdCAM is a ligand found on mucosal endothelial cells to direct cells into inflamed tissues [106]. Alpha5 beta1 binds fibronectin and osteopontin. 

The roots of deciduous teeth are resorbed physiologically during normal tooth. Pathophysiologic root resorption usually involves resorption of the roots of permanent teeth [107]. One example is root resorption associated with orthodontic force application [108]. During orthodontic procedures a small amount of root resorption often occurs, but not enough to cause short- or long-term difficulties with tooth retention. In some patients, for reasons that are not clear, the application of mechanical force triggers massive root resorption that endangers the health and retention of the tooth. Although dentine and bone are both mineralized matrices with similar compositions, our study showed that there are subtle differences in the response of the osteoclasts to bone versus dentine. A crucial difference was the shedding of RANK-containing EVs, which occurred at lower levels from osteoclasts resorbing dentine compared with bone [25].

The difference in the predominant integrins being shed may also be illuminating. AlphaM/L/X beta2 binds fibrinogen, Factor X, iC3b, and intercellular adhesion molecules, which are soluble ligands associated with inflammation or cell surface receptors of cell-cell interactions [109]. It is possible that the beta2 integrin containing EVs could play anti-inflammatory roles by competitively inhibiting interactions between pro-inflammatory ligands with the integrins on the cell. The relative abundance of alpha4 in EVs from osteoclasts resorbing dentine, could support the idea that these EVs may be acting in an anti-inflammatory manner, competitively inhibiting integrin interactions that are involved in inflammatory responses. AlphaV beta3 is important for osteoclast activation and binds denatured collagen, like that present during bone resorption [110,111,112,113,114]. It will be of great interest to determine if alphaV beta3 integrins are found in EVs that also carry RANK. If so, this could be a mechanism for targeting the RANK EVs to the sites where new bone formation is required. We believe that studies to determine the composition of EVs with different integrins are of vital importance. We hypothesize that specific EVs will have one type of integrin and that regulatory factors will be associated with specific EVs.

**Figure 6:**
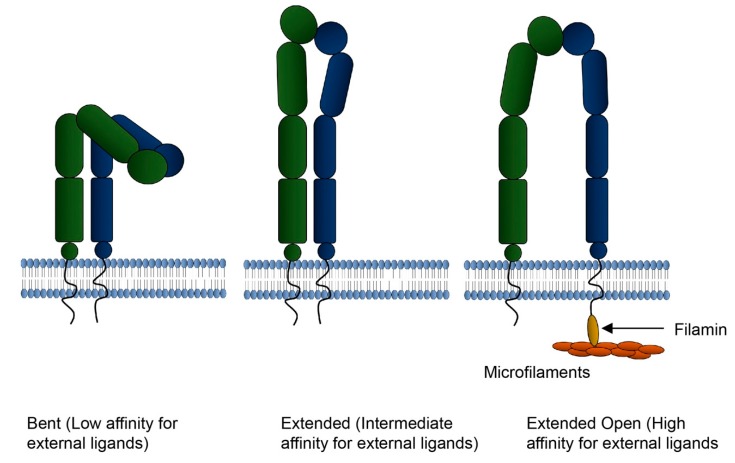
Integrins can take on multiple conformations. In the bent conformation the integrin does not bind external ligands. In extended conformation, integrins bind ligands with intermediate affinity. In the extended open conformation integrins bind ligands with high affinity. Attachment of talin to the cytosolic (luminal) domain of the beta3 integrin can promote the high affinity bonding conformation. In the schematic, alpha integrins are on the left, beta integrins are on right. As described in text, filamin and other integrin–associated proteins can regulate the conformation of integrins by associating with the cytosolic domain.

As described above, there is considerable evidence for integrins targeting EVs to specific locations in the organism. Integrins serve as an information conduit. When they bind an external ligand, not only does it serve, in some cases, as a means to secure adhesion with the extracellular matrix, or in some cases, another cell, but it also triggers signaling within the cell. A complex of proteins that bind the cytosolic region of the integrin accumulate, recruit the actin cytoskeleton and trigger signal transduction pathways [115]. Integrins also serve as a conduit for inside-out signaling [116,117,118]. To bind external ligands with high affinity, the integrin must be in a specific conformation. This is accomplished by the binding of specific actin-associated proteins (Figure 6). For integrins in EVs to bind extracellular matrix, as has been described, it must be in a conformation that enables that binding, and that is achieved by the interaction with proteins including filamin. Likewise, other binding proteins of the cytosolic (luminal) domain of integrins can lock them into a conformation where they cannot bind their ligands in the outside world.

For EVs to utilize integrins for targeting, they must also have elements necessary to maintain them in their high affinity binding conformation. Our proteomic analysis of osteoclast EVs suggests that the proteins necessary to maintain active integrins are present, and so the idea of a membrane cytoskeleton of actin-associated proteins in EVs is plausible. Experiments are crucial to identify the binding state of integrins in EVs and the overall compositions of those EVs. Affinity isolation of the EVs using the integrin-ligand interaction has been accomplished for EVs from B-cells [92].

When the fusion of EVs with target cells is considered, it is usually thought of as delivering luminal components, like microRNAs, to the cytosol of the target cell [119,120]. Another consequence of the fusion of EVs is the delivery of membrane components, including integrins [121,122]. In a series of studies, data has been presented that suggests that EVs deliver integrins from a cell of origin to a target cell, and that this is an element of the machinery that allows cancer cells to precondition the local environment of a site of metastasis [93,121]. With osteoclast EVs, both alphaV beta3 and alpha2 beta1 integrins have been shown to have critical regulatory functions in osteoblasts [123,124,125]. It is plausible that fusion of osteoclast EVs, containing these integrins with osteoblasts could enhance signaling responses that are intrinsic to osteoblasts. This is particularly intriguing in the scenario where integrins first attach EVs, with signal like RANK on their surface, to areas where bone has been resorbed, then contact and fuse with osteoblasts. This could both stimulate osteoblastogenesis through reverse RANKL signaling and enhance the response by donating integrins that would increase adherence and adherence based signaling in the osteoblasts.

## 8. Summary

Actin and various actin-associated proteins are among the most abundant components that make up EVs, including EVs shed by osteoclasts. It is not known whether these proteins are present because they were part of the mechanism by which EVs are made, or whether they are involved in the regulatory function of EVs. Although EVs likely lack sufficient ATP to support dynamic filaments, and key elements of the machinery that stimulate actin polymerization are missing, it is possible that as EVs fuse with target cells, and the incorporation of concentrated cytoskeletal components could enhance cellular responses like directed cell movement. Integrins have been reported to be vital for targeting EVs to the extracellular matrix. Actin-associated proteins, and perhaps microfilaments in EVs may function to maintain integrins in the conformation that binds the extracellular matrix tightly. EVs have only recently emerged as important agents of intercellular signaling. Understanding the roles of the abundant actin cytoskeletal proteins is crucial for the long-term goal of fully understanding signaling by EVs and making use of that understanding to develop new therapeutic approaches.

## Abbreviations

EV	Extracellular vesicle
RANK	Receptor activator of nuclear factor kappa B
RANKL	Receptor activator of nuclear factor kappa B-ligand
ESCRT	Endosomal sorting complexes required for transport
ATP	Adenosine triphosphate
ADP-Pi	Adenosine diphosphate with attached phosphate group
ADP	Adenosine diphosphate
WASP	Wiskott-Aldrich syndrome protein
Mical	Molecule interacting with CasL
MadCAM	Mucosal addressin cell adhesion molecule
ITGB	Integrin beta
ITGA	Integrin alpha
MYH	Myosin heavy chain
MYO	Myosin
ACTN	Alpha actinin
TAGLN	Transgelin
CAP	Capping protein

## References

[1] Mathieu M., Martin-Jaular L., Lavieu G., Thery C. (2019). Specificities of secretion and uptake of exosomes and other extracellular vesicles for cell-to-cell communication. Nat. Cell Biol..

[2] Tkach M., Thery C. (2016). Communication by Extracellular Vesicles: Where We Are and Where We Need to Go. Cell.

[3] Witwer K.W., Thery C. (2019). Extracellular vesicles or exosomes? On primacy, precision, and popularity influencing a choice of nomenclature. J. Extracell. Vesicles.

[4] Raposo G., Stoorvogel W. (2013). Extracellular vesicles: Exosomes, microvesicles, and friends. J. Cell Biol..

[5] Zhang H., Freitas D., Kim H.S., Fabijanic K., Li Z., Chen H., Mark M.T., Molina H., Martin A.B., Bojmar L. (2018). Identification of distinct nanoparticles and subsets of extracellular vesicles by asymmetric flow field-flow fractionation. Nat. Cell Biol..

[6] Harding C., Heuser J., Stahl P. (1983). Receptor-mediated endocytosis of transferrin and recycling of the transferrin receptor in rat reticulocytes. J. Cell Biol..

[7] Pan B.T., Johnstone R.M. (1983). Fate of the transferrin receptor during maturation of sheep reticulocytes in vitro: Selective externalization of the receptor. Cell.

[8] Raposo G., Nijman H.W., Stoorvogel W., Liejendekker R., Harding C.V., Melief C.J., Geuze H.J. (1996). B lymphocytes secrete antigen-presenting vesicles. J. Exp. Med..

[9] Valadi H., Ekstrom K., Bossios A., Sjostrand M., Lee J.J., Lotvall J.O. (2007). Exosome-mediated transfer of mRNAs and microRNAs is a novel mechanism of genetic exchange between cells. Nat. Cell Biol..

[10] Deregibus M.C., Cantaluppi V., Calogero R., Lo Iacono M., Tetta C., Biancone L., Bruno S., Bussolati B., Camussi G. (2007). Endothelial progenitor cell derived microvesicles activate an angiogenic program in endothelial cells by a horizontal transfer of mRNA. Blood.

[11] Li D., Liu J., Guo B., Liang C., Dang L., Lu C., He X., Cheung H.Y., Xu L., Lu C. (2016). Osteoclast-derived exosomal miR-214-3p inhibits osteoblastic bone formation. Nat. Commun..

[12] Sun W., Zhao C., Li Y., Wang L., Nie G., Peng J., Wang A., Zhang P., Tian W., Li Q. (2016). Osteoclast-derived microRNA-containing exosomes selectively inhibit osteoblast activity. Cell Discov..

[13] Chevillet J., Kang Q., Ruf I.K., Briggs H.A., Vojtech L.N., Hughes S.M., Cheng H.H., Arroyo J.D., Meredith E.K., Gallichotte E.N. (2014). Quantitative and stoichiometric analysis of the microRNA content of exosomes. Proc. Natl. Acad. Sci. USA.

[14] Huynh N., VonMoss L., Smith D., Rahman I., Felemban M.F., Zuo J., Rody W.J., McHugh K.P., Holliday L.S. (2016). Characterization of regulatory extracellular vesicles from osteoclasts. J. Dent. Res..

[15] Negishi-Koga T., Shinohara M., Komatsu N., Bito H., Kodama T., Friedel R.H., Takayanagi H. (2011). Suppression of bone formation by osteoclastic expression of semaphorin 4D. Nat. Med..

[16] Takyar F.M., Tonna S., Ho P.W., Crimeen-Irwin B., Baker E.K., Martin T.J., Sims N.A. (2013). EphrinB2/EphB4 inhibition in the osteoblast lineage modifies the anabolic response to parathyroid hormone. J. Bone Miner. Res..

[17] Boyce B.F., Xing L. (2008). Functions of RANKL/RANK/OPG in bone modeling and remodeling. Arch. Biochem. Biophys..

[18] Hofbauer L.C., Kuhne C.A., Viereck V. (2004). The OPG/RANKL/RANK system in metabolic bone diseases. J. Musculoskelet. Neuronal Interact..

[19] Holliday L.S., McHugh K.P., Zuo J., Aguirre J.I., Neubert J.K., Rody W.J. (2017). Exosomes: Novel regulators of bone remodeling and potential therapeutic agents for orthodontics. Orthod. Craniofac. Res..

[20] Ikebuchi Y., Aoki S., Honma M., Hayashi M., Sugamori Y., Khan M., Kariya Y., Kato G., Tabata Y., Penninger J.M. (2018). Coupling of bone resorption and formation by RANKL reverse signalling. Nature.

[21] (2012). Sclerostin regulates RANKL expression in osteocytes. Bonekey Rep..

[22] Killock D. (2011). Bone: Osteocyte RANKL in bone homeostasis: A paradigm shift?. Nat. Rev. Rheumatol..

[23] Nakashima T., Hayashi M., Fukunaga T., Kurata K., Oh-Hora M., Feng J.Q., Bonewald L.F., Kodama T., Wutz A., Wagner E.F. (2011). Evidence for osteocyte regulation of bone homeostasis through RANKL expression. Nat. Med..

[24] Xiong J., Onal M., Jilka R.L., Weinstein R.S., Manolagas S.C., O’Brien C.A. (2011). Matrix-embedded cells control osteoclast formation. Nat. Med..

[25] Rody W.J., Chamberlain C.A., Emory-Carter A.K., McHugh K.P., Wallet S.M., Spicer V., Krokhin O., Holliday L.S. (2019). The proteome of extracellular vesicles released by clastic cells differs based on their substrate. PLoS ONE.

[26] Teitelbaum S.L. (2007). Osteoclasts: What do they do and how do they do it?. Am. J. Pathol..

[27] Chambers T.J., Fuller K. (2011). How are osteoclasts induced to resorb bone?. Ann. N. Y. Acad. Sci..

[28] Destaing O., Petropoulos C., Albiges-Rizo C. (2014). Coupling between acto-adhesive machinery and ECM degradation in invadosomes. Cell Adh. Migr..

[29] King G.J., Holtrop M.E. (1975). Actin-like filaments in bone cells of cultured mouse calvaria as demonstrated by binding to heavy meromyosin. J. Cell Biol..

[30] Blair H.C., Teitelbaum S.L., Ghiselli R., Gluck S. (1989). Osteoclastic bone resorption by a polarized vacuolar proton pump. Science.

[31] Toro E.J., Ostrov D.A., Wronski T.J., Holliday L.S. (2012). Rational Identification of Enoxacin as a Novel V-ATPase-Directed Osteoclast Inhibitor. Curr. Protein Pept. Sci..

[32] Bromme D., Okamoto K., Wang B.B., Biroc S. (1996). Human cathepsin O_2_, a matrix protein-degrading cysteine protease expressed in osteoclasts. Functional expression of human cathepsin O_2_ in Spodoptera frugiperda and characterization of the enzyme. J. Biol. Chem..

[33] Gelb B.D., Moissoglu K., Zhang J., Martignetti J.A., Bromme D., Desnick R.J. (1996). Cathepsin K: Isolation and characterization of the murine cDNA and genomic sequence, the homologue of the human pycnodysostosis gene. Biol. Chem. Mol. Med..

[34] Tomoshige S., Kobayashi Y., Hosoba K., Hamamoto A., Miyamoto T., Saito Y. (2017). Cytoskeleton-related regulation of primary cilia shortening mediated by melanin-concentrating hormone receptor 1. Gen. Comp. Endocrinol..

[35] Collier M.E., Maraveyas A., Ettelaie C. (2014). Filamin-A is required for the incorporation of tissue factor into cell-derived microvesicles. Thromb. Haemost..

[36] Collier M.E.W., Ettelaie C., Goult B.T., Maraveyas A., Goodall A.H. (2017). Investigation of the Filamin A-Dependent Mechanisms of Tissue Factor Incorporation into Microvesicles. Thromb. Haemost..

[37] Colombo M., Moita C., Van Niel G., Kowal J., Vigneron J., Benaroch P., Manel N., Moita L.F., Thery C., Raposo G. (2013). Analysis of ESCRT functions in exosome biogenesis, composition and secretion highlights the heterogeneity of extracellular vesicles. J. Cell Sci..

[38] Ostrowski M., Carmo N.B., Krumeich S., Fanget I., Raposo G., Savina A., Moita C.F., Schauer K., Hume A.N., Freitas R.P. (2010). Rab27a and Rab27b control different steps of the exosome secretion pathway. Nat. Cell Biol..

[39] Trajkovic K., Hsu C., Chiantia S., Rajendran L., Wenzel D., Wieland F., Schwille P., Brugger B., Simons M. (2008). Ceramide triggers budding of exosome vesicles into multivesicular endosomes. Science.

[40] Yuyama K., Sun H., Mitsutake S., Igarashi Y. (2012). Sphingolipid-modulated exosome secretion promotes clearance of amyloid-beta by microglia. J. Biol. Chem..

[41] Pollard T.D. (2017). What We Know and Do Not Know About Actin. Handb. Exp. Pharmacol..

[42] Kajimoto T., Mohamed N.N.I., Badawy S.M.M., Matovelo S.A., Hirase M., Nakamura S., Yoshida D., Okada T., Ijuin T., Nakamura S.I. (2018). Involvement of Gβγ subunits of G(i) protein coupled with S1P receptor on multivesicular endosomes in F-actin formation and cargo sorting into exosomes. J. Biol. Chem..

[43] Footer M.J., Kerssemakers J.W., Theriot J.A., Dogterom M. (2007). Direct measurement of force generation by actin filament polymerization using an optical trap. Proc. Natl. Acad. Sci. USA.

[44] DePina A.S., Langford G.M. (1999). Vesicle transport: The role of actin filaments and myosin motors. Microsc. Res. Technol..

[45] Hurley J.H. (2015). ESCRTs are everywhere. EMBO J..

[46] Devreotes P.N., Bhattacharya S., Edwards M., Iglesias P.A., Lampert T., Miao Y. (2017). Excitable Signal Transduction Networks in Directed Cell Migration. Ann. Rev. Cell Dev. Biol..

[47] Hammer J.A. (1994). The structure and function of unconventional myosins: A review. J. Muscle Res. Cell Motil..

[48] Wang K., Wloka C., Bi E. (2019). Non-muscle Myosin-II Is Required for the Generation of a constriction Site for Subsequent Abscission. iScience.

[49] Muralidharan-Chari V., Clancy J., Plou C., Romao M., Chavrier P., Raposo G., D’Souza-Schorey C. (2009). ARF6-regulated shedding of tumor cell-derived plasma membrane microvesicles. Curr. Biol.

[50] Sun W., Vida T.A., Sirisaengtaksin N., Merrill S.A., Hanson P.I., Bean A.J. (2010). Cell-free reconstitution of multivesicular body formation and receptor sorting. Traffic.

[51] Carlier M.F., Wiesner S., Le C.C., Pantaloni D. (2003). Actin-based motility as a self-organized system: Mechanism and reconstitution In Vitro. Comptes Rendus Biol..

[52] Theriot J.A., Rosenblatt J., Portnoy D.A., Goldschmidt-Clermont P.J., Mitchison T.J. (1994). Involvement of profilin in the actin-based motility of L. monocytogenes in cells and in cell-free extracts. Cell.

[53] Korn E.D. (1982). Actin polymerization and its regulation by proteins from nonmuscle cells. Physiol. Rev..

[54] Campellone K.G., Welch M.D. (2010). A nucleator arms race: Cellular control of actin assembly. Nat. Rev. Mol. Cell Biol..

[55] Lasser C., Jang S.C., Lotvall J. (2018). Subpopulations of extracellular vesicles and their therapeutic potential. Mol. Aspects Med..

[56] Zabeo D., Cvjetkovic A., Lasser C., Schorb M., Lotvall J., Hoog J.L. (2017). Exosomes purified from a single cell type have diverse morphology. J. Extracell. Vesicles..

[57] Korn E.D., Carlier M.F., Pantaloni D. (1987). Actin polymerization and ATP hydrolysis. Science.

[58] Ronquist K.G., Ek B., Morrell J., Stavreus-Evers A., Strom H.B., Humblot P., Ronquist G., Larsson A. (2013). Prostasomes from four different species are able to produce extracellular adenosine triphosphate (ATP). Biochim. Biophys. Acta.

[59] Guo H., Chang Z., Zhang Z., Zhao Y., Jiang X., Yu H., Zhang Y., Zhao R., He B. (2019). Extracellular ATPs produced in seminal plasma exosomes regulate boar sperm motility and mitochondrial metabolism. Theriogenology.

[60] Pantaloni D., Carlier M.F. (1993). How profilin promotes actin filament assembly in the presence of thymosin beta 4. Cell.

[61] Zhang Q., Higginbotham J.N., Jeppesen D.K., Yang Y.P., Li W., McKinley E.T., Graves-Deal R., Ping J., Britain C.M., Dorsett K.A. (2019). Transfer of Functional Cargo in Exomeres. Cell Rep..

[62] Lal A.A., Brenner S.L., Korn E.D. (1984). Preparation and polymerization of skeletal muscle ADP-actin. J. Biol. Chem..

[63] Zigmond S.H. (2004). Formin-induced nucleation of actin filaments. Curr. Opin. Cell Biol..

[64] Rotty J.D., Wu C., Bear J.E. (2013). New insights into the regulation and cellular functions of the ARP2/3 complex. Nat. Rev. Mol. Cell Biol..

[65] Machesky L.M., Gould K.L. (1999). The Arp2/3 complex: A multifunctional actin organizer. Curr. Opin. Cell Biol..

[66] Machesky L.M., Atkinson S.J., Ampe C., Vandekerckhove J., Pollard T.D. (1994). Purification of a cortical complex containing two unconventional actins from Acanthamoeba by affinity chromatography on profilin-agarose. J. Cell Biol..

[67] Millard T.H., Machesky L.M. (2001). The Wiskott-Aldrich syndrome protein (WASP) family. Trends Biochem. Sci..

[68] Weaver A.M., Heuser J.E., Karginov A.V., Lee W.L., Parsons J.T., Cooper J.A. (2002). Interaction of cortactin and N-WASp with Arp2/3 complex. Curr. Biol..

[69] Luxenburg C., Parsons J.T., Addadi L., Geiger B. (2006). Involvement of the Src-cortactin pathway in podosome formation and turnover during polarization of cultured osteoclasts. J. Cell Sci..

[70] Tehrani S., Faccio R., Chandrasekar I., Ross F.P., Cooper J.A. (2006). Cortactin has an essential and specific role in osteoclast actin assembly. Mol. Biol. Cell.

[71] Zalli D., Neff L., Nagano K., Shin N.Y., Witke W., Gori F., Baron R. (2016). The Actin-Binding Protein Cofilin and Its Interaction with Cortactin Are Required for Podosome Patterning in Osteoclasts and Bone Resorption In Vivo and In Vitro. J. Bone Miner. Res..

[72] Chan K.T., Creed S.J., Bear J.E. (2011). Unraveling the enigma: Progress towards understanding the coronin family of actin regulators. Trends Cell Biol..

[73] Blanchoin L., Amann K.J., Higgs H.N., Kaiser D.A., Marchand J.B., Mullins R.D., Pollard T.D. (2000). Role of ADF/cofilin, Arp2/3 complex, capping proteins and profilin in the dynamic of branched actin filaments networks. Mol. Biol. Cell.

[74] Blanchoin L., Pollard T.D., Mullins R.D. (2000). Interactions of ADF/cofilin, Arp2/3 complex, capping protein and profilin in remodeling of branched actin filament networks. Curr. Biol..

[75] Blanchoin L., Mullins R.D., Robinson R.C., Choe S., Pollard T.D. (1999). Acanthamoeba actophorin (ADF/cofilin) depolymerizes actin filaments capped with Arp2/3 and gelsolin as well as only barbed ends capped filaments. Effect of phosphorylation on actophorin interaction with actin. Mol. Biol. Cell.

[76] Sung B.H., Ketova T., Hoshino D., Zijlstra A., Weaver A.M. (2015). Directional cell movement through tissues is controlled by exosome secretion. Nat. Commun..

[77] Sung B.H., Weaver A.M. (2017). Exosome secretion promotes chemotaxis of cancer cells. Cell Adhes. Migr..

[78] Zhou Y., Gunput R.A., Adolfs Y., Pasterkamp R.J. (2011). MICALs in control of the cytoskeleton, exocytosis, and cell death. Cell Mol. Life Sci..

[79] Grintsevich E.E., Ge P., Sawaya M.R., Yesilyurt H.G., Terman J.R., Zhou Z.H., Reisler E. (2017). Catastrophic disassembly of actin filaments via Mical-mediated oxidation. Nat. Commun..

[80] Cooper J.A., Schafer D.A. (2000). Control of actin assembly and disassembly at filament ends. Curr. Opin. Cell Biol..

[81] Bearer E.L., Prakash J.M., Manchester R.D., Allen P.G. (2000). VASP protects actin filaments from gelsolin: An in vitro study with implications for platelet actin reorganizations. Cell Motil. Cytoskeleton..

[82] Christensen J.R., Hocky G.M., Homa K.E., Morganthaler A.N., Hitchcock-DeGregori S.E., Voth G.A., Kovar D.R. (2017). Competition between Tropomyosin, Fimbrin, and ADF/Cofilin drives their sorting to distinct actin filament networks. eLife.

[83] Gateva G., Kremneva E., Reindl T., Kotila T., Kogan K., Gressin L., Gunning P.W., Manstein D.J., Michelot A., Lappalainen P. (2017). Tropomyosin Isoforms Specify Functionally Distinct Actin Filament Populations In Vitro. Curr. Biol..

[84] Na B.R., Jun C.D. (2015). TAGLN2-mediated actin stabilization at the immunological synapse: Implication for cytotoxic T cell control of target cells. BMB Rep..

[85] Na B.R., Kim H.R., Piragyte I., Oh H.M., Kwon M.S., Akber U., Lee H.S., Park D.S., Song W.K., Park Z.Y. (2015). TAGLN2 regulates T cell activation by stabilizing the actin cytoskeleton at the immunological synapse. J. Cell Biol..

[86] Hao R., Liu Y., Du Q., Liu L., Chen S., You H., Dong Y. (2019). Transgelin-2 expression in breast cancer and its relationships with clinicopathological features and patient outcome. Breast Cancer.

[87] Sun Y., Peng W., He W., Luo M., Chang G., Shen J., Zhao X., Hu Y. (2018). Transgelin-2 is a novel target of KRAS-ERK signaling involved in the development of pancreatic cancer. J. Exp. Clin. Cancer Res..

[88] Zhou Q., Jiang X., Yan W., Dou X. (2019). Transgelin 2 overexpression inhibits cervical cancer cell invasion and migration. Mol. Med. Rep..

[89] Vinterhoj H.S.H., Stensballe A., Duroux M., Gazerani P. (2019). Characterization of rat primary trigeminal satellite glial cells and associated extracellular vesicles under normal and inflammatory conditions. J. Proteom..

[90] Krits I., Wysolmerski R.B., Holliday L.S., Lee B.S. (2002). Differential Localization of Myosin II Isoforms in Resting and Activated Osteoclasts. Calcif. Tissue Int..

[91] Zhang Y., Cao F., Zhou Y., Feng Z., Sit B., Krendel M., Yu C.H. (2019). Tail domains of myosin-1e regulate phosphatidylinositol signaling and F-actin polymerization at the ventral layer of podosomes. Mol. Biol. Cell.

[92] Clayton A., Turkes A., Dewitt S., Steadman R., Mason M.D., Hallett M.B. (2004). Adhesion and signaling by B cell-derived exosomes: The role of integrins. FASEB J..

[93] DeRita R.M., Sayeed A., Garcia V., Krishn S.R., Shields C.D., Sarker S., Friedman A., McCue P., Molugu S.K., Rodeck U. (2019). Tumor-Derived Extracellular Vesicles Require beta1 Integrins to Promote Anchorage-Independent Growth. iScience.

[94] Guo Q., Furuta K., Lucien F., Gutierrez Sanchez L.H., Hirsova P., Krishnan A., Kabashima A., Pavelko K.D., Madden B., Alhuwaish H. (2019). Integrin beta1-enriched extracellular vesicles mediate monocyte adhesion and promote liver inflammation in murine NASH. J. Hepatol..

[95] Manou D., Caon I., Bouris P., Triantaphyllidou I.E., Giaroni C., Passi A., Karamanos N., Vigetti D., Theocharis A.D. (2019). The Complex Interplay between Extracellular Matrix and Cells in Tissues. Methods Mol. Biol..

[96] Hoshino D., Kirkbride K.C., Costello K., Clark E.S., Sinha S., Grega-Larson N., Tyska M.J., Weaver A.M. (2013). Exosome secretion is enhanced by invadopodia and drives invasive behavior. Cell Rep..

[97] Genschmer K.R., Russell D.W., Lal C., Szul T., Bratcher P.E., Noerager B.D., Abdul R.M., Xu X., Rezonzew G., Viera L. (2019). Activated PMN Exosomes: Pathogenic Entities Causing Matrix Destruction and Disease in the Lung. Cell.

[98] Chen W., Hoffmann A.D., Liu H., Liu X. (2018). Organotropism: New insights into molecular mechanisms of breast cancer metastasis. NPJ Precis. Oncol..

[99] Hoshino A., Costa-Silva B., Shen T.L., Rodrigues G., Hashimoto A., Tesic M.M., Molina H., Kohsaka S., Di G.A., Ceder S. (2015). Tumour exosome integrins determine organotropic metastasis. Nature.

[100] Harburger D.S., Calderwood D.A. (2009). Integrin signalling at a glance. J. Cell Sci..

[101] Hayashi H., Nakahama K., Sato T., Tuchiya T., Asakawa Y., Maemura T., Tanaka M., Morita I. (2008). The role of Mac-1 (CD11b/CD18) in osteoclast differentiation induced by receptor activator of nuclear factor-kappaB ligand. FEBS Lett..

[102] Ohtsuji M., Lin Q., Okazaki H., Takahashi K., Amano H., Yagita H., Nishimura H., Hirose S. (2018). Anti-CD11b antibody treatment suppresses the osteoclast generation, inflammatory cell infiltration, and autoantibody production in arthritis-prone FcgammaRIIB-deficient mice. Arthritis Res. Ther..

[103] Yang G., Chen X., Yan Z., Zhu Q., Yang C. (2017). CD11b promotes the differentiation of osteoclasts induced by RANKL through the spleen tyrosine kinase signalling pathway. J. Cell Mol. Med..

[104] Hamzei M., Ventriglia G., Hagnia M., Antonopolous A., Bernal-Sprekelsen M., Dazert S., Hildmann H., Sudhoff H. (2003). Osteoclast stimulating and differentiating factors in human cholesteatoma. Laryngoscope.

[105] Ruef N., Dolder S., Aeberli D., Seitz M., Balani D., Hofstetter W. (2017). Granulocyte-macrophage colony-stimulating factor-dependent CD11c-positive cells differentiate into active osteoclasts. Bone.

[106] Strauch U.G., Lifka A., Gosslar U., Kilshaw P.J., Clements J., Holzmann B. (1994). Distinct binding specificities of integrins alpha 4 beta 7 (LPAM-1), alpha 4 beta 1 (VLA-4), and alpha IEL beta 7. Int. Immunol..

[107] Ne R.F., Witherspoon D.E., Gutmann J.L. (1999). Tooth resorption. Quintessence Int..

[108] Hartsfield J.K. (2009). Pathways in external apical root resorption associated with orthodontia. Orthod. Craniofac. Res..

[109] Humphries J.D., Byron A., Humphries M.J. (2006). Integrin ligands at a glance. J. Cell Sci..

[110] Chellaiah M.A., Hruska K.A. (2003). The integrin alpha(v)beta(3) and CD44 regulate the actions of osteopontin on osteoclast motility. Calcif. Tissue Int..

[111] Duong L.T., Lakkakorpi P., Nakamura I., Rodan G.A. (2000). Integrins and signaling in osteoclast function. Matrix Biol..

[112] McHugh K.P., Hodivala-Dilke K., Zheng M.H., Namba N., Lam J., Novack D., Feng X., Ross F.P., Hynes R.O., Teitelbaum S.L. (2000). Mice lacking beta 3 integrins are osteosclerotic because of dysfunctional osteoclasts. J. Clin. Investig..

[113] McHugh K.P., Shen Z., Crotti T.N., Flannery M.R., Fajardo R., Bierbaum B.E., Goldring S.R. (2007). Role of cell-matrix interactions in osteoclast differentiation. Adv. Exp. Med. Biol..

[114] Holliday L.S., Welgus H.G., Fliszar C.J., Veith G.M., Jeffrey J.J., Gluck S.L. (1997). Initiation of osteoclast bone resorption by interstitial collagenase. J. Biol. Chem..

[115] Horton E.R., Humphries J.D., James J., Jones M.C., Askari J.A., Humphries M.J. (2016). The integrin adhesome network at a glance. J. Cell Sci..

[116] Faull R.J., Ginsberg M.H. (1996). Inside-out signaling through integrins. J. Am. Soc. Nephrol..

[117] Shen B., Delaney M.K., Du X. (2012). Inside-out, outside-in, and inside-outside-in: G protein signaling in integrin-mediated cell adhesion, spreading, and retraction. Curr. Opin. Cell Biol..

[118] Springer T.A., Dustin M.L. (2012). Integrin inside-out signaling and the immunological synapse. Curr. Opin. Cell Biol..

[119] Bayraktar R., Van R.K., Calin G.A. (2017). Cell-to-cell communication: microRNAs as hormones. Mol. Oncol..

[120] Kim K.M., Abdelmohsen K., Mustapic M., Kapogiannis D., Gorospe M. (2017). RNA in extracellular vesicles. Wiley Interdiscip. Rev. RNA.

[121] Fedele C., Singh A., Zerlanko B.J., Iozzo R.V., Languino L.R. (2015). The alphavbeta6 integrin is transferred intercellularly via exosomes. J. Biol. Chem..

[122] Singh A., Fedele C., Lu H., Nevalainen M.T., Keen J.H., Languino L.R. (2016). Exosome-mediated Transfer of alphavbeta3 Integrin from Tumorigenic to Nontumorigenic Cells Promotes a Migratory Phenotype. Mol. Cancer Res..

[123] Cheng S.L., Lai C.F., Fausto A., Chellaiah M., Feng X., McHugh K.P., Teitelbaum S.L., Civitelli R., Hruska K.A., Ross F.P. (2000). Regulation of alphaVbeta3 and alphaVbeta5 integrins by dexamethasone in normal human osteoblastic cells. J. Cell Biol. Chem..

[124] Hu H.M., Yang L., Wang Z., Liu Y.W., Fan J.Z., Fan J., Liu J., Luo Z.J. (2013). Overexpression of integrin a2 promotes osteogenic differentiation of hBMSCs from senile osteoporosis through the ERK pathway. Int. J. Clin. Exp. Pathol..

[125] Phillips J.A., Almeida E.A., Hill E.L., Aguirre J.I., Rivera M.F., Nachbandi I., Wronski T.J., van der Meulen M.C., Globus R.K. (2008). Role for beta1 integrins in cortical osteocytes during acute musculoskeletal disuse. Matrix Biol..

